# Brunner’s Gland Hyperplasia Causing Complete Small Bowel Obstruction in a Patient With Helicobacter pylori

**DOI:** 10.7759/cureus.41351

**Published:** 2023-07-04

**Authors:** Toni-Ann J Lewis, Sofya Kostanyan, Franklin Kasmin

**Affiliations:** 1 Internal Medicine, New York-Presbyterian Brooklyn Methodist Hospital, Brooklyn, USA; 2 Gastroenterology, New York-Presbyterian Brooklyn Methodist Hospital, Brooklyn, USA

**Keywords:** helicobacter pylori, brunner’s gland hamartoma, gastrointestinal obstruction, brunner's gland hyperplasia, small bowel obstruction

## Abstract

Brunner's gland hyperplasia is an uncommon pathology from the duodenum and is believed to be associated with infection with *Helicobacter pylori*. Patients commonly present with gastrointestinal bleeding, nausea, or abdominal pain. However, obstruction is an unusual clinical finding.

A 47-year-old male presented to the emergency department with complaints of recurrent emesis, epigastric pain, and cramping for three days. Medical history was significant for duodenitis and diverticulitis, but there had been no prior abdominal surgeries. Epigastric tenderness to palpation without rebound tenderness was present on physical examination, *H. pylori* stool antigen was positive on admission, and treatment with triple therapy was initiated. Progressively the patient developed increasing emesis, with an associated cessation in flatus and bowel movements. On endoscopy, it was reported that the endoscope could not advance past the second portion of the duodenum. A nasogastric tube was placed for gastric decompression. Small bowel follow-through showed obstruction at the distal second duodenal segment. Bismuth quadruple therapy was initiated on day three. Push enteroscopy showed luminal narrowing and a transition point at the second duodenal segment with no identifiable mass or significant ulceration. Biopsy reports indicated Brunner's gland hyperplasia. By day seven, the patient reported increased bowel movements and flatus, with a resolution of his nausea and emesis, and the nasogastric tube was removed. The patient was discharged on day eight with outpatient prescriptions for quadruple therapy for six days. He was also instructed to follow up with the general surgery and gastroenterology teams for outpatient colonoscopy six weeks post-discharge and with his primary care physician (PCP) four weeks after completing quadruple therapy to ensure *H. pylori *eradication.

Studies have shown that *H. pylori* were detected in most patients with Brunner's gland hyperplasia and may induce proliferation in Brunner's glands. Brunner's gland hyperplasia has a low incidence, with minimal cases reported. There is malignant potential but a low risk of progression into adenocarcinoma. Our case reinforces the idea that Brunner's gland hyperplasia should be included in the work-up, alongside testing for infection with *H. pylori* in assessing patients with gastric obstruction.

## Introduction

Brunner's gland hyperplasia, an unusual lesion from the duodenum, makes up 10.6% of all duodenal tumors [1]. In most cases, these lesions are benign and infrequently necessitate surgical intervention. It has been studied that infection with *Helicobacter pylori *can cause Brunner's gland hyperplasia, although this association remains mainly unknown [2]. The initial joint patient presentation is gastrointestinal bleeding, nausea, or abdominal pain. Frequently patients are asymptomatic, and lesions are discovered incidentally via endoscopy. Gastric obstruction, however, is an unusual clinical finding [3].

## Case presentation

**Figure 1:**
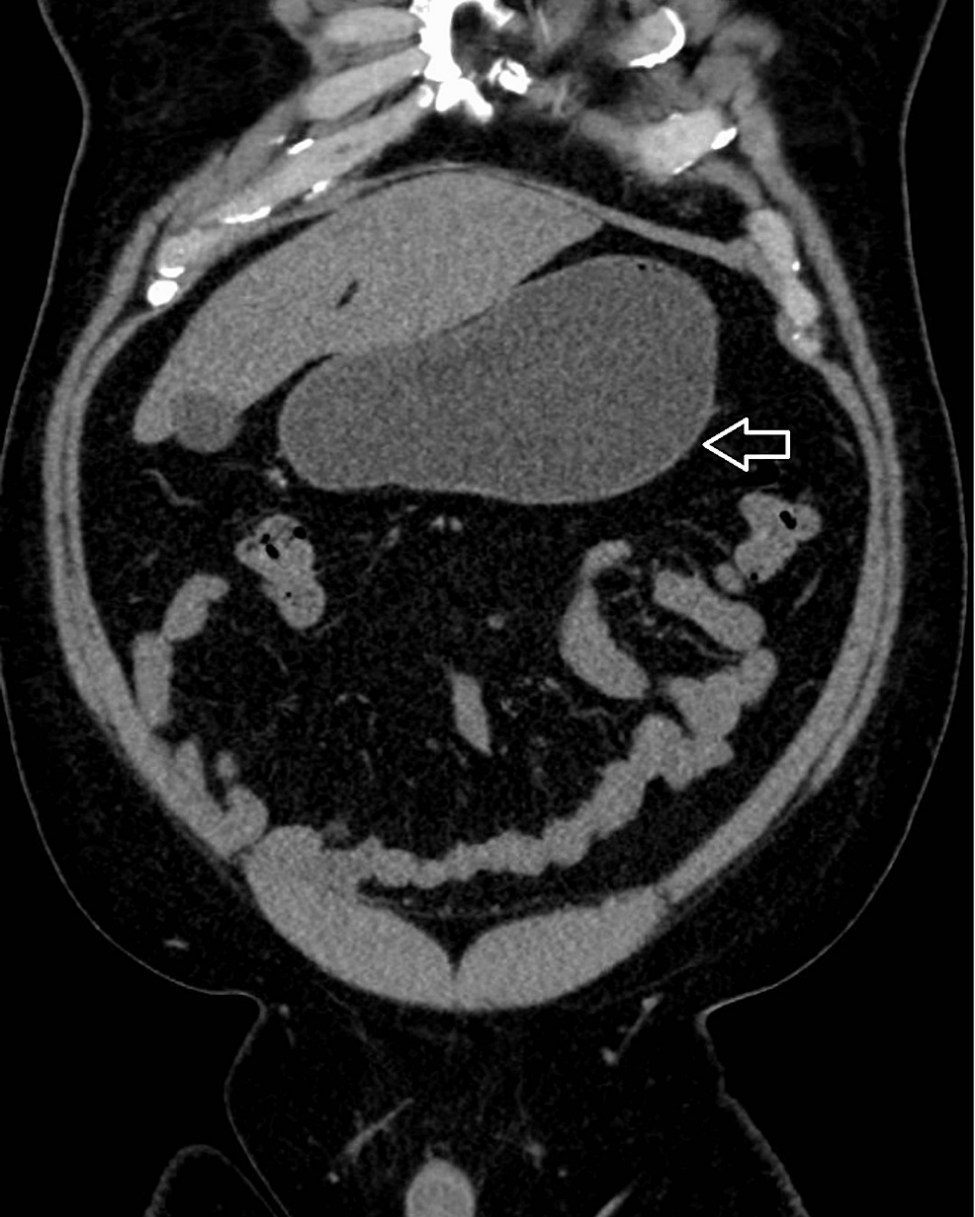
CT abdomen pelvis coronal view showing stomach distention (arrow)

A 47-year-old male presented to the emergency department with complaints of recurrent intractable large-volume non-bloody bilious emesis, with associated epigastric pain and cramping for three days. Medical record was significant for duodenitis and diverticulitis, but no prior abdominal surgeries were recorded. Upon physical examination, there was epigastric tenderness to palpation without rebound tenderness. *H. pylori* stool antigen was positive on admission. Intravenous treatment of *H. pylori* with ciprofloxacin 500 mg, metronidazole 500 mg, and pantoprazole 40 mg was initiated. Progressively, this patient developed increasing emesis, with an associated decrease in flatus and bowel movements. A CT abdomen and pelvis from prior admission showed a distended stomach/duodenal bulb with fat stranding around the duodenum and hyper-density within the proximal duodenum and stomach (Figure 1). On subsequent endoscopy, the day of admission, it was reported that the endoscope could not advance past the second portion of the duodenum.

**Figure 2:**
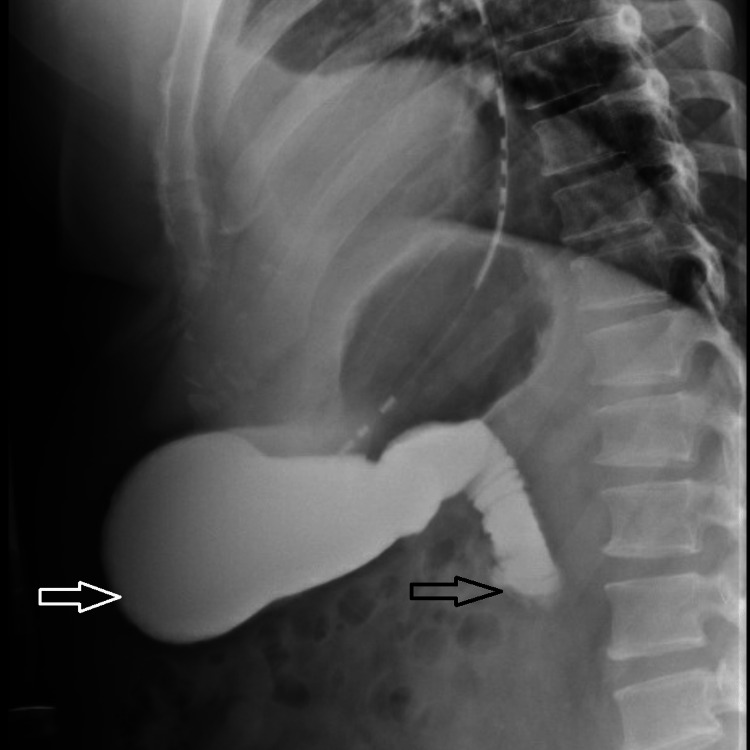
Upper gastrointestinal series showing stomach distention (white arrow) past the second portion of duodenum (black arrow)

**Figure 3:**
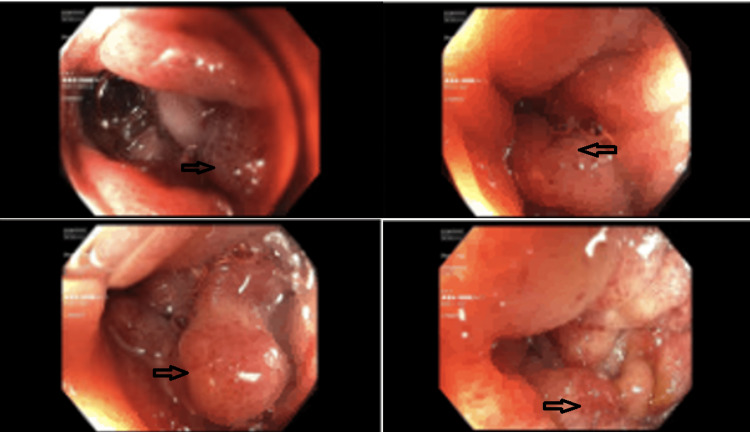
Endoscopic findings showing abnormal mucosa (arrows)

**Figure 4:**
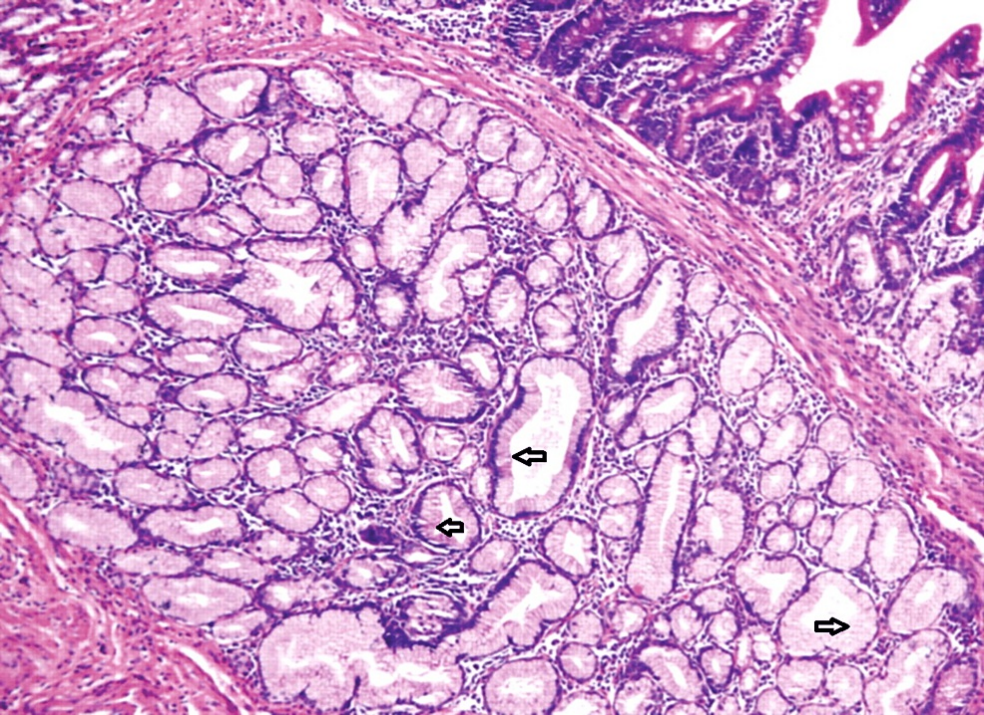
Benign uniform columnar cells (arrows) seen on high powered view suggestive of Brunner’s gland hyperplasia

Due to his increasing emesis and abdominal distention, there was a high suspicion of bowel obstruction. A nasogastric tube was placed and set to low intermittent suction with a daily output of over 500 mL. The patient endorsed no bowel movements or flatus episodes, and an abdominal examination revealed no bowel sounds. Metabolic derangements were noted during this period, likely due to recurrent emesis, and electrolytes were supplemented as indicated. Small bowel follow-through showed obstruction at the distal second duodenal segment (Figure 2). On day three of admission, bismuth quadruple therapy was initiated to attenuate inflammation and discomfort from *H. Pylori*. Push enteroscopy showed luminal narrowing and a transition point at the second duodenal segment with no identifiable mass or significant ulceration (Figure 3). Biopsy reports indicated Brunner's gland hyperplasia (Figure 4). By day seven, the patient reported increased bowel movements and flatus, with a resolution of his nausea and emesis, and, therefore, the nasogastric tube was removed. The diet was advanced and tolerated. The patient was discharged on day eight with outpatient prescriptions for quadruple therapy for six days. He was also instructed to follow up with the general surgery and gastroenterology teams for outpatient colonoscopy six weeks post-discharge and with his primary care physician (PCP) for *H. pylori* stool antigen four weeks after completing quadruple therapy to ensure eradication.

## Discussion

Small bowel obstruction counts for relatively 300,000 inpatient admissions in the United States, most of which are related to adhesions from prior surgery (approximately 70%) [4]. Our patient's clinical presentation, alongside various endoscopic and imaging techniques, revealed complete small bowel obstruction. However, he lacked a pertinent history of abdominopelvic surgery or other known risk factors. Additionally, imaging and laboratory work-up revealed no evidence of congenital malformations, abnormally ingested materials, or alimentary canal abnormalities. The most pertinent in this patient's medical history were diverticulitis, duodenitis, and the incidentally found *H. pylori* on admission. It was discovered through a biopsy that he was positive for Brunner's gland hyperplasia.

Brunner's glands are duodenal glands localized primarily within the submucosa of the proximal duodenum. These glands secrete an alkaline substance containing mucin (Muc-6), which protects the duodenal lining by neutralizing the acid from the stomach [3]. It is theorized that excess gastric acid excretion or increased inflammation may cause hyperplasia in these glands [1]. Brunner's gland hyperplasia becomes more infrequent past the proximal duodenum: 57% found within the duodenal bulb, 27% found within the second portion of the duodenum, 7% found within the third portion of the duodenum, 2% within the jejunum and terminal ileum, and 5% found on the pylorus [5]. Treatment is typically resection via endoscopy, laparoscopy, or laparotomy [6].

Brunner's gland hyperplasia is usually an incidental endoscopic finding in most asymptomatic patients [3]. Depending on location and size, patients may present with nonspecific symptoms like nausea, bloating, abdominal pain, anemia, and gastric obstruction on rare occasions [3]. These lesions are rare, often benign, and have an estimated incidence of 0.008%, with fewer than 200 cases reported [1,3]. The majority of these cases were resolved with surgical intervention. Two cases by Lu Li et al. reported patients with Brunner's gland hamartoma: one initially presented complaining of melena with associated anemia, and the other with complaints of fever and leukopenia [7]. Both patients were hemodynamically stable on presentation, underwent endoscopic imaging that revealed a duodenal mass, both taken for successful exploratory laparotomy and did well following the procedure. Another case of Brunner's hamartoma reported by Park et al. had an initial presentation similar to ours [8]. Their patient had a three-day episode of nausea, vomiting, and epigastric pain; small bowel series revealed a lobular filling defect within the jejunum, and the mass was removed via successful laparoscopic enterotomy. Alternatively, in our case, in spite of presenting with obstruction, the patient did not require surgical intervention and did well with medical management alone.

The association between Brunner's gland hyperplasia and* H. pylori *is minimally studied, and it is believed that* H. pylori *infection could also be involved in its pathology [2]. Studies have reported that during the autopsy, *H. pylori *were identified in 71% of patients with Brunner's gland hyperplasia and that *H. pylori* may induce proliferation in Brunner's glands [2]. Other reports suggest that* H. pylori* infections and gastritis may induce the progression of an existing hyperplastic lesion through chronic irritation and injury to the duodenal mucosa [2,9]. The most contributing aspects studied in mucosal injury are increased gastric acid secretion, duodenal mechanical stimuli, and* H. pylori* infections [2]. An identical study evaluated the association between Brunner's glands and* H. pylori* and found gastritis and duodenitis in 61.54% and 84.62% of patients, respectively [2]. It was also found that there have been* H. pylori *within the gastric mucosa, and 66.7% of these had Brunner's gland lesions after duodenal biopsy [2]. These lesions are usually benign; however, they have malignant potential with a frequency of less than 1% among the primary tumors of the small intestine [9].

## Conclusions

In summary, we presented an unusual case of small bowel obstruction caused by Brunner's gland hyperplasia, likely induced by chronic duodenitis and exacerbated by infection with *H. pylori*. Our patient's presenting symptoms were likely relieved through mechanical decompression from push enteroscopy and quadruple therapy. The patient did not require surgical intervention and found relief with medical management. These factors make our case distinct from others found in the literature. Our case reinforces the idea that in assessing patients with gastric obstruction, a seemingly benign case of Brunner's gland hyperplasia should be included in the work-up, alongside testing for infection with* H. pylori*.
